# Comparison of the Effectiveness and Safety of Segmental Thoracic Spinal Anesthesia Using Isobaric Levobupivacaine 0.5% Versus Hyperbaric Levobupivacaine 0.5% in Performing Laparoscopic Cholecystectomy: A Prospective Randomized Controlled Trial

**DOI:** 10.7759/cureus.76060

**Published:** 2024-12-20

**Authors:** Anil K Verma, Neha Kumar, Chaitra Srinivas, Priyanka Sahu

**Affiliations:** 1 Anesthesiology, Ganesh Shankar Vidyarthi Memorial (GSVM) Medical College, Kanpur, IND; 2 Anesthesiology and Critical Care, Ganesh Shankar Vidyarthi Memorial (GSVM) Medical College, Kanpur, IND; 3 Anesthesiology, Cardiac Care Unit (CCU) Fortis Hospital, Lucknow, IND; 4 Community Medicine, Autonomous State Medical College, Auraiya, IND

**Keywords:** hemodynamic stability, hyperbaric anaesthesia, laparoscopic cholecystectomy, levobupivacaine, thoracic segmental spinal anaesthesia

## Abstract

Introduction: Laparoscopic cholecystectomy has evolved into a daycare procedure thanks to advancements in both surgical and anesthetic techniques. Regional anesthesia, specifically segmental thoracic spinal anesthesia (TSA), offers distinct benefits over general anesthesia, such as enhanced hemodynamic stability and quicker recovery, especially in high-risk patients. This study aims to compare the sensory and motor block characteristics, hemodynamic stability, and incidence of adverse effects between isobaric and hyperbaric 0.5% levobupivacaine in segmental TSA for laparoscopic cholecystectomy.

Methodology: A prospective, randomized, double-blind trial was conducted from May to August 2024 at GSVM Medical College, Kanpur. A total of 60 patients, classified as American Society of Anesthesiologists (ASA) I and II, scheduled to undergo elective laparoscopic cholecystectomy, were randomly assigned to two groups, with 30 patients in each group. This randomization process was conducted after obtaining ethical approval and registering the study with the Clinical Trials Registry of India (CTRI). Group B received 1.5 mL of hyperbaric 0.5% levobupivacaine with 25 mcg fentanyl via TSA at the T10-T11 interspace, while Group A received 1.5 mL of isobaric 0.5% levobupivacaine with 25 mcg fentanyl. Various parameters, including hemodynamic changes, adverse effects, satisfaction scores, maximum sensory block height, and the onset and duration of both sensory and motor blocks, were recorded. Postoperative pain was assessed using the visual analog scale (VAS).

Results: Group B demonstrated higher levels of sensory and motor block, with a faster onset, leading to superior surgical conditions and higher patient satisfaction scores. Group A, on the other hand, not only experienced a longer block duration but also reported more negative side effects, including bradycardia and hypotension, which led to higher postoperative discomfort. Hemodynamic analysis showed that throughout the early time points (2-8 minutes), Group A had a considerably lower heart rate and systolic and diastolic blood pressure.

Conclusions: Hyperbaric levobupivacaine provided faster block onset and offset, improved satisfaction, better hemodynamic stability, and quicker recovery. It is a safe and effective anesthetic choice for laparoscopic cholecystectomy, offering predictable block spread and fewer adverse effects compared to isobaric levobupivacaine.

## Introduction

Segmental spinal anesthesia for laparoscopic cholecystectomy involves administering a local anesthetic to block specific spinal segments to provide sufficient anesthesia for the upper abdomen [1]. This technique targets selected dermatomes, allowing for effective pain relief during the procedure while minimizing motor block and preserving respiratory function. Unlike traditional general anesthesia (GA), segmental spinal anesthesia reduces the risk of hemodynamic instability, such as fluctuations in blood pressure and heart rate, and offers a quicker recovery. It is particularly beneficial in high-risk patients, where GA may be less suitable due to underlying medical conditions. The procedure typically requires a smaller dose of anesthetic, as the thoracic spinal region, with its lower cerebrospinal fluid (CSF) volume and thinner nerve roots, requires less drug to achieve the desired block [1,2].

Laparoscopic procedures like cholecystectomy have become a daycare procedure due to advancements in surgical as well as anesthesia techniques [3,4] and lumbar spinal anesthesia [5] has been an emerging technique, especially in cases with significant medical problems and in those with a high risk of GA [4,6]. GA has its downsides like high hemodynamic variations, side effects of anesthetic drugs, delayed recovery, postoperative pain, and occasionally dealing with difficult airways [4]. Regional anesthesia is preferred with advancements in techniques and the availability of new drugs. Thoracic segmental spinal anesthesia is on the verge of development with greater efficacy and safety in procedures involving the upper abdomen and thoracic regions [6]. Segmental spinal refers to blocking selected dermatomes for surgical procedures with low but effective doses [7]. Since the thoracic thecal space has a lower CSF volume and the thoracic roots are thinner, less drug is needed to block the dermatomes. Since the dose is low, there will be less sympathetic block and fewer hemodynamic variations Through MRI of the spinal cord structure, Imbelloni showed that the thoracic area had a higher space between the posterior chord and the dura mater, mostly because of thoracic kyphosis. The distance between the spinal needle tip and the spinal cord is further increased by the 45-degree angle required for spinal needle implantation due to the oblique alignment of the thoracic vertebrae [8,9]. Sitting with the head down position further increases the thoracic curve. All these points are in favor of a low prevalence of paresthesias with thoracic puncture. Respiration is not hampered as the diaphragm is spared as it is supplied by C3, C4, and C5, and intercostal muscles are not affected as the motor block is less. The T1-T4 segments are the source of the cardioaccelerator fibers, and severe bradycardia may result from a high neural block. However, since the lumbosacral segments are undamaged, blood cannot pool in the lower limbs, making this less likely to happen. Consequently, the right atrium's fullness is maintained [10].

Regional anesthesia in the space between T10 and L1 is adequate for all abdominal surgeries, eliminating the necessity for thoracic spinal above T10. Effective anesthesia lasts for 90 to 120 minutes when 7.5 to 10 mg (1.5-2 mL) of bupivacaine or levobupivacaine is used with adjuncts such as fentanyl or clonidine. This dosage is about half of what is required for lumbar-level conventional spinal anesthesia to produce a T3-T4 block. Levobupivacaine 0.5%, the S-enantiomer of bupivacaine, is less hazardous than its racemic counterpart. This medication, which comes in both isobaric and hyperbaric versions, has a gradual onset, aids in hemodynamic stability, promotes early bladder function recovery, and has a shorter period of motor block for faster movement.

Levobupivacaine, a widely used local anesthetic, has been studied in different formulations, with isobaric solutions having the same density as CSF and hyperbaric solutions being denser. These variations impact the drug's spread within the subarachnoid space, influencing sensory and motor block characteristics. Isobaric solutions tend to provide more predictable and uniform effects, whereas hyperbaric solutions, due to their increased density, may result in a more concentrated block at specific spinal levels. By comparing the two, clinicians can better tailor anesthetic choices for procedures such as spinal anesthesia, aiming for optimal efficacy and safety that is why we compare both the drugs. Differential neuraxial block preserving motor function at low concentrations provides an advantage to levobupivacaine.

There are few studies comparing levobupivacaine with bupivacaine in thoracic segmental spinal anesthesia. Levobupivacaine's isobaric and hyperbaric versions have only been examined in one study, and there is currently insufficient evidence to determine which is better. For elective laparoscopic cholecystectomy, thoracic segmental spinal anesthesia offers a safe and efficient method with favorable operating conditions [11,12]. When used carefully, this method can be a useful substitute for GA, particularly in circumstances where the latter may not be suitable. Shoulder tip pain is a common complaint during laparoscopic cholecystectomy (lap chole) due to the insufflation of gas (usually carbon dioxide) into the abdomen. This technique, when used carefully, can be a useful substitute for GA, particularly in circumstances where the latter may not be suitable.

The primary objective of this study was to compare the effects of isobaric and hyperbaric 0.5% levobupivacaine on sensory and motor blocks during segmental thoracic spinal anesthesia (TSA) for laparoscopic cholecystectomy. The secondary objectives included comparing the impact of isobaric and hyperbaric 0.5% levobupivacaine on hemodynamic stability, particularly blood pressure and heart rate, throughout the procedure. Additionally, the study aimed to evaluate and compare the incidence of side effects, such as low blood pressure, slow heart rate, or headaches, between the two types of levobupivacaine.

## Materials and methods

Study design and ethical approval

**Figure 1:**
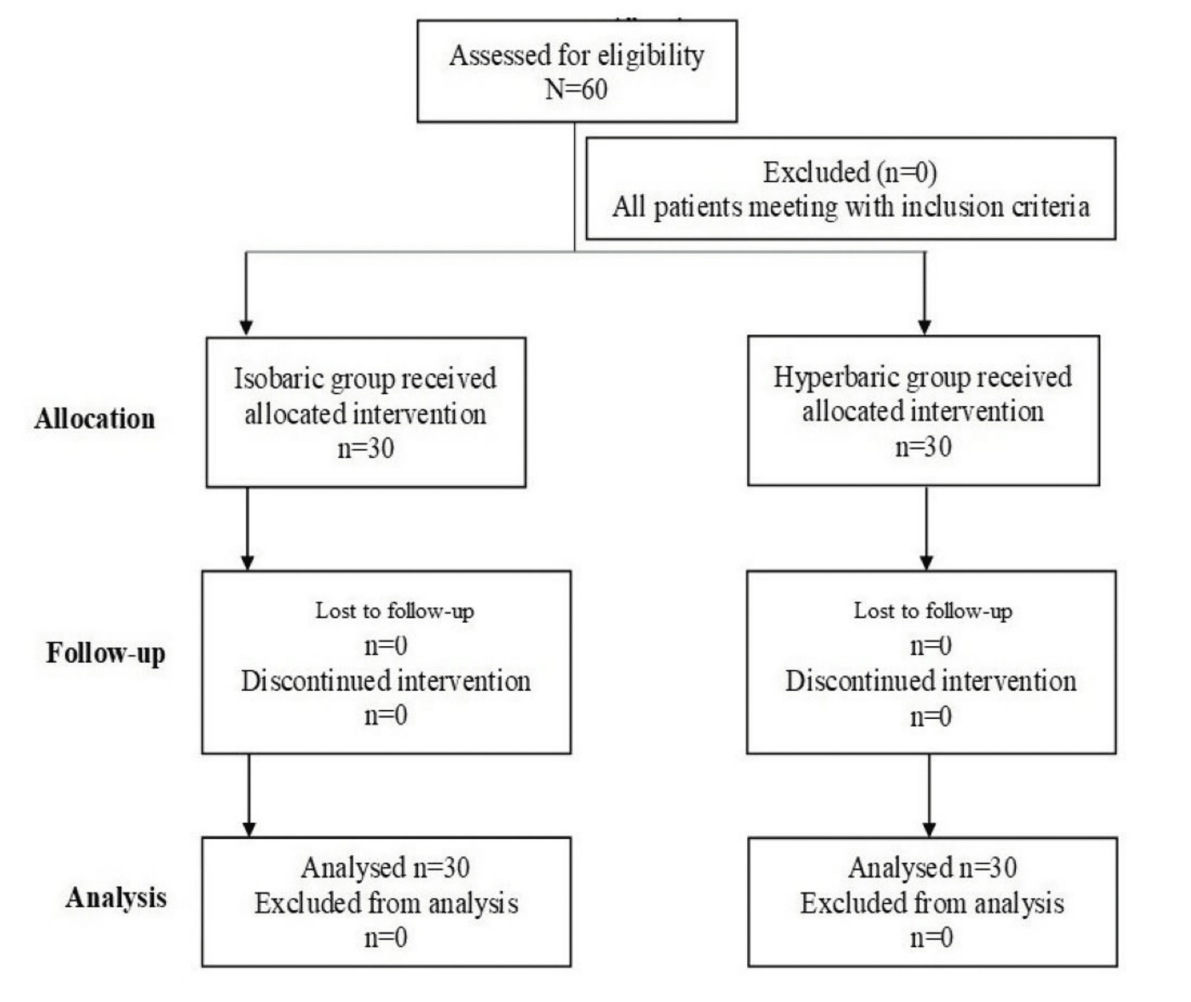
CONSORT diagram. CONSORT, Consolidated Standards of Reporting Trials

The institutional ethics committee approved this prospective, randomized, double-blind, comparative study under reference number EC/BMHR/2024/117, dated February 26, 2024. The study was conducted in the Department of Anesthesia, GSVM Medical College, and the associated LLRH Hospital in Kanpur from May to August 2024. It involved patients scheduled for elective laparoscopic cholecystectomy. Additionally, the trial was registered with the Clinical Trials Registry of India (CTRI) under the registration number CTRI/2024/09/073241. Randomization was done by computer-generated numbers. The anesthesiologist who gave spinal anesthesia was blinded, and the observer was also blinded (Figure 1).

Sample size

The sample size was calculated based on the onset of the motor block:

μ1 = 6.13

S1 = 1.86

μ2 = 5.02

S2 = 1.15

n = (S1^2 + S2^2) * (Zα/2 + Z1-p)^2 / (μ1 - μ2)^2

= {(1.86)^2 + (1.15)^2} * (1.96 + 0.84)^2 / (6.13 - 5.02)^2

= (3.46^2 + 1.32) * (2.8)^2 / (1.11)^2

= (4.78) * (7.84) / (1.11)^2

= 37.47 / 1.23

n = 30.46

Inclusion and exclusion criteria

The inclusion criteria for the study were patients aged 18 to 60 years who provided informed written consent, with the American Society of Anesthesiologists (ASA) classifications I and II, and scheduled for elective laparoscopic cholecystectomy. Patients had to have a surgery duration not exceeding 90 minutes. The exclusion criteria included patients who did not provide consent, those with ASA classifications III or IV, a body mass index (BMI) greater than 30 kg/m², a history of obstructive sleep apnea, anticipated difficult intubation, acute cholecystitis or pancreatitis, neuromuscular or neuropsychiatric diseases, spine deformities or previous spine surgery, coagulation abnormalities, local infections, contraindications to regional anesthesia, and allergies to local anesthetics.

Study subjects

Written consent was obtained from all participants. The patients were then randomly assigned into two equal groups using computer-generated random numbers. Thirty patients were included in Group A and 30 in Group B. Before the surgery, all patients received 150 mg of Tab Rantac and 0.5 mg of Tab Alprax. Fasting guidelines were followed, with no solid food for eight hours, no liquids for four hours, and no clear liquids for two hours before the procedure. Patients were informed about the possibility of discomfort or pain during the surgery, with immediate intervention available if necessary, including the potential use of supplemental GA.

Anesthesia technique and monitoring

Standard ASA monitoring was used, including non-invasive blood pressure (NIBP), pulse oximetry (SpO2), end-tidal CO2 (ETCO2), and electrocardiogram (ECG). An 18 G intravenous cannula was placed, and for preloading, 15 mL/kg of Ringer lactate was administered over 30 minutes. Intravenous Ondansetron (0.1 mg/kg) and Ranitidine Hydrochloride (50 mg) were given as pre-medication.

The T10-T11 interspace was located in the sitting position by identifying T7, which corresponds to the inferior angle of the scapula. After local anesthesia administration, spinal anesthesia was performed using a 25 G Quincke needle with CSF confirmation.

Group assignments and drug administration

In Group A, 1.5 mL of isobaric 0.5% levobupivacaine with 25 mcg of fentanyl was administered. In Group B, 1.5 mL of hyperbaric 0.5% levobupivacaine with 25 mcg of fentanyl was used. The patient was placed in a supine position (lying flat) without any tilt, and oxygen was delivered at 5 L/minute through a face mask.

Assessment of sensory and motor block

To check the sensory block, a pinprick test was performed along the midclavicular line on both sides, and the time to reach sensory block levels at T4 and L2 was recorded. The highest level of sensory block was noted. The motor block onset was checked using the modified Bromage scale (grade 1), and the time to reach the maximum motor block level was also recorded.

The Modified Bromage Scale was used to assess the motor block by evaluating the patient's ability to move their lower extremities. A score of 0 indicates that the patient can lift their leg straight and move the hip, knee, and ankle. A score of 1 means the patient cannot lift the leg straight but can still flex the knee and ankle freely. A score of 2 shows that the patient is unable to flex the knee and hip but can flex the ankle. A score of 3 means the patient cannot flex the ankle, knee, or hip, but can still move their toes. A score of 4 indicates that there is no movement in the lower extremity.

Intraoperative management

ECG, heart rate, blood pressure, SpO2, respiratory rate, and temperature were monitored every 2 minutes for the first 15 minutes, and then every 5 minutes thereafter. Once the desired block level was achieved, surgery was initiated with CO2 insufflation at a pressure limit of 12 mmHg and minimal table tilt. Additionally, 10 mL of 2% lignocaine was sprayed under the right dome of the diaphragm.

Management of adverse events

Any adverse events during the procedure were promptly addressed. For pain (right shoulder or abdominal pain), Inj Butrum 2 mg IV was administered. For anxiety, Inj Midazolam 0.05 mg/kg IV was given. Bradycardia was treated with Inj Atropine 0.01 mg/kg IV, and hypotension was managed with a fluid bolus of 10 ml/kg, followed by Inj Efipress 6 mg IV bolus (up to 30 mg). Nausea and vomiting were treated with Inj Ondansetron 0.04 mg/kg, and shivering was addressed with Inj Tramadol 50 mg. Pruritus and respiratory depression were closely monitored, and the surgery was switched to GA if necessary.

Duration of blocks and postoperative care

The duration of the sensory block was recorded from the onset at the T4 dermatome to when it regressed to the T10 dermatome. The duration of the motor block was noted from the time of maximum block until the patient regained the ability to raise extended legs (Bromage scale 0).

The Patient Discomfort Score was evaluated at 10-minute intervals during surgery and 15 minutes postoperatively, ranging from 0 (none) to 3 (severe). Patient Satisfaction Scores were recorded on a 0 to 5 scale, with 1 point given for the absence of specific complaints such as postoperative pain, awareness during surgery, postoperative nausea/vomiting, postoperative urinary retention, or headache/backache. Similarly, the Surgeon Satisfaction Score, also on a 0 to 5 scale, assessed the absence of pain during surgery, intraoperative movements, need for sedation or GA conversion, postoperative side effects, and timely discharge from the hospital.

Postoperative pain assessment

Postoperative pain was assessed using the visual analog scale (VAS) score from 0 to 10 at 0, 1, 2, 6, 12, and 24 hours after surgery. Rescue analgesia used included Inj Tramadol 100 mg plus Inj Paracetamol 1 g. Adverse effects, including nausea, vomiting, shivering, urinary retention, headache, shoulder pain, backache, and neurological symptoms (such as paresthesia), were observed and compared between both groups.

Statistical analysis

The data were recorded in a Microsoft Excel sheet and analyzed using SPSS version 24 (IBM Corp., Armonk, NY). The normality of the data was assessed, and categorical variables were presented as frequencies and percentages. To compare the two groups, the chi-square test and independent t-test were used for categorical and parametric data, respectively, while a paired t-test was employed to calculate the delta mean. A *P*-value of less than 0.05 was considered statistically significant.

## Results

The results showed that Group B had a faster onset and stronger sensory and motor blocks, resulting in better surgical outcomes and higher patient satisfaction. On the other hand, Group A experienced a longer period of sensory and motor block along with a higher frequency of side effects, such as hypotension and bradycardia. Furthermore, Group A had significantly higher postoperative discomfort and a lower level of satisfaction. These findings suggest that while Group B had faster and more effective block characteristics, Group A might have benefitted from longer block durations (Table 1).

**Table 1:** Clinical and demographic characteristics of the study population. Isobaric 0.5% levobupivacaine was used as Group A, and hyperbaric 0.5% levobupivacaine was used as Group B. The chi-square test and Student's t-test were used to calculate the *P*-value. ^*^P < 0.05 was considered as statistically significant. BMI, body mass index; ASA, American Society of Anesthesiologist - physical status; Max, maximum; Min, minimum; Postop, postoperative; VAS, visual analog scale

Variables	Group A (*N *= 30), *n* (%)	Group B (*N *= 30), *n* (%)	*P*-value
Age (years), mean ± SD	39.10 ± 10.55	40.47 ± 9.67	0.602
Gender			
Male	28 (93.3)	27 (90.0)	0.640
Female	2 (6.7)	3 (10.0)	
ASA grade			
Grade 1	22 (73.3)	19 (63.3)	0.405
Grade 2	8 (26.7)	11 (36.7)	
Heart rate, mean ± SD (Baseline)	97.07 ± 12.68	103.77 ± 11.61	0.037*
Systolic, mean ± SD (Baseline)	126.43 ± 12.48	127.43 ± 12.61	0.758
Diastolic, mean ± SD (Baseline)	86.47 ± 10.45	87.00 ± 11.68	0.853
Onset of sensory block (Min), mean ± SD	2.10 ± 0.25	1.06 ± 0.13	<0.001*
Time to T4 (Min), mean ± SD	2.74 ± 0.48	1.45 ± 0.13	<0.001*
Max height of the sensory block			
T3	12 (40.0)	0 (0.0)	0.635
T4	18 (60.0)	30 (100.0)	
Duration of the sensory block (Min), mean ± SD	189.17 ± 22.09	170.00 ± 7.43	<0.001*
Onset of the motor block (Min), mean ± SD	5.02 ± 0.44	1.48 ± 0.14	<0.001*
Max Bromage scale			
2	14 (46.7)	0 (0.0)	0.34
3	15 (50.0)	4 (13.3)	
4	1 (3.3)	26 (86.7)	
Time to reach the max motor block (Min)	5.66 ± 0.44	2.15 ± 0.22	<0.001*
Duration of the motor block (Min), mean ± SD	162.67 ± 13.69	135.33 ± 12.24	<0.001*
Adverse effect			
Bradycardia	5 (16.7)	0 (0.0)	0.324
Hypotension	9 (30.0)	1 (3.3)	
NIL	16 (53.3)	29 (96.7)	
Sedation (antianxiety) used			
Yes	14 (46.7)	12 (40.0)	0.670
No	16 (53.3)	18 (60.0)	
Analgesic			
Yes	14 (46.7)	1 (3.3)	<0.001*
No	16 (53.3)	29 (96.7)	
SpO2 (%), mean ± SD	98.77 ± 0.77	98.57 ± 1.01	0.391
Duration of surgery (Min), mean ± SD	35.33 ± 7.06	29.17 ± 8.62	0.003*
Surgeon satisfactory score			
3	6 (20.0)	1 (3.3)	0.008*
4	9 (30.0)	3 (10.0)	
5	15 (50.0)	26 (86.7)	
Patient satisfaction score			
4	14 (46.7)	4 (13.3)	0.004*
5	16 (53.3)	26 (86.7)	
Discomfort score			
Yes	14 (46.7)	1 (3.3)	<0.001*
No	16 (53.3)	29 (96.7)	
Postoperative pain (VAS), 1 hour			
0	18 (60.0)	24 (80.0)	0.756
1	9 (30.0)	6 (20.0)	
2	3 (10.0)	-	
Mean ± SD	0.00 ± 0.00	0.21 ± 0.41	0.012*
Postop VAS 2 hours			
0	17 (56.7)	17 (56.7)	0.342
1	1 (3.3)	7 (23.3)	
2	5 (16.7)	1 (3.3)	
3	7 (23.3)	3 (10.0)	
4	-	2 (6.7)	
Mean ± SD	0.00 ± 0.00	0.86 ± 0.13	0.012*
Time for rescue analgesia (minutes), mean ± SD	199.33 ± 22.04	187.00 ± 9.52	0.466

**Figure 2:**
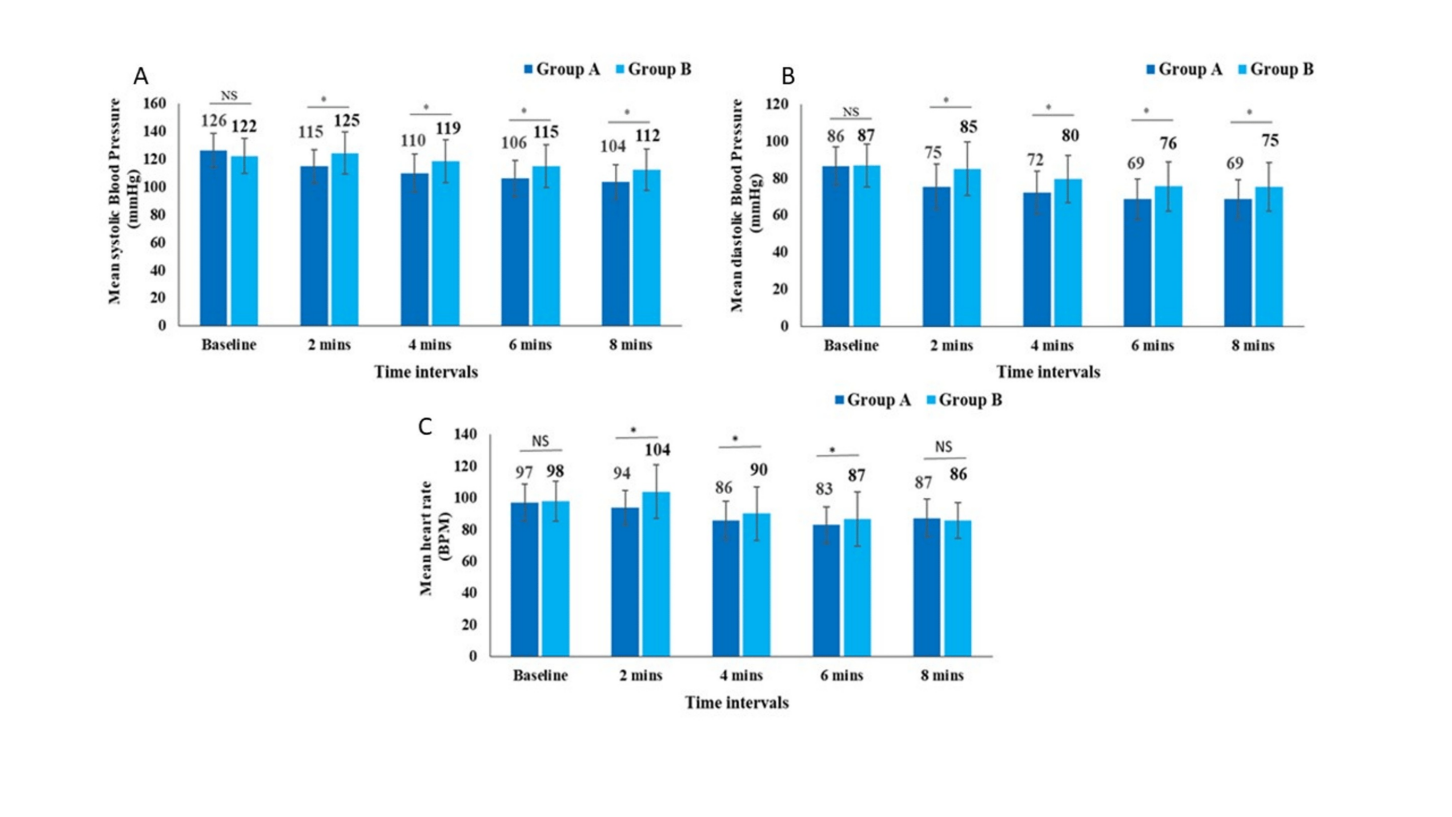
Status of (A) systolic, (B) diastolic blood pressure, and (C) heart rate at different time intervals. The X-axis represents the time intervals, and the Y-axis represents the mean levels of the variables. An asterisk (*) indicates a *P*-value of < 0.05.

The diastolic, systolic, and heart rate conditions at various time intervals are shown in Figure 2. According to the systolic and diastolic blood pressure (DBP) statistics, Group A's blood pressure was significantly lower than that of Group B during the 2- to 8-minute interval (*P *< 0.05).

Significant variations were seen in heart rates between Group A and Group B at 2, 4, and 6 minutes after the intervention. The baseline heart rates of the groups did not differ significantly (Δ mean = 2.89, *P* = 0.414). In contrast to Group A, Group B's heart rate increased significantly more at 2 minutes (Δ mean = 10.01, *P* = 0.005). During the early post-intervention phase, Group B's heart rate showed a sharper increase, with this trend continuing at 4 and 6 minutes (Δ mean = 4.00, *P* = 0.024 and Δ mean = 5.01, *P* = 0.017, respectively). Heart rate differences appeared to have resolved by the time the groups were 8 minutes apart (Δ mean = -1.47, *P* = 0.568) (Table 2).

**Table 2:** Average heart rate variation across all study groups according to time interval. Isobaric 0.5% levobupivacaine was used as Group A, and hyperbaric 0.5% levobupivacaine was used as Group B. The paired t-test was applied to calculate the mean differences (delta mean). ^*^P < 0.05 was considered statistically significant. BPM, beats per minute

Heart rate (BPM)	Group A vs. Group B	
Time intervals	*r* mean	*P*-value
Baseline	2.89	0.414
2 minutes	10.01	0.005*
4 minutes	4.00	0.024*
6 minutes	5.01	0.017*
8 minutes	-1.47	0.568

Table 3 shows that the comparison of systolic blood pressure (SBP) between Group A and Group B revealed significant differences at 2, 4, 6, and 8 minutes post-intervention. The groups' SBPs did not differ significantly at baseline (Δ mean = 4.00, *P* = 0.784). However, Group B's SBP increased significantly more than Group A's at 2 minutes (Δ mean = 9.81, *P* = 0.001). At 4 minutes (Δ mean = 8.83, *P* = 0.018), 6 minutes (Δ mean = 8.71, *P* = 0.013), and 8 minutes (Δ mean = 7.93, *P* = 0.037), this difference was still significant.

**Table 3:** Mean difference in systolic blood pressure between all the study groups based on time intervals. Isobaric 0.5% levobupivacaine was used as Group A, and hyperbaric 0.5% levobupivacaine was used as Group B. The paired t-test was applied to calculate the mean differences (delta mean). ^*^P < 0.05 was considered statistically significant.

SBP (mmHg)	Group A vs. Group B	
Time intervals	*r* mean	*P*-value
Baseline	4.00	0.784
2 minutes	9.81	0.001*
4 minutes	8.83	0.018*
6 minutes	8.71	0.013*
8 minutes	7.93	0.037*

The comparison of DBP between Group A and Group B revealed significant differences at 2, 4, 6, and 8 minutes post-intervention. DBP did not significantly differ across the groups at baseline (Δ mean = 0.533, *P* = 0.847). However, Group B's DBP increased much more than Group A's at 2 minutes (Δ mean = 9.66, *P* = 0.009). This trend continued at 4 minutes (Δ mean = 7.36, *P* = 0.029), 6 minutes (Δ mean = 6.96, *P* = 0.036), and 8 minutes (Δ mean = 6.70, *P* = 0.032), with Group B consistently showing higher DBP values (Table 4).

**Table 4:** Mean difference in diastolic blood pressure (DBP) between all the study groups based on time intervals. Isobaric 0.5% levobupivacaine was used for Group A, and hyperbaric 0.5% levobupivacaine was used for Group B. The paired t-test was used to calculate the mean differences (delta mean). ^*^P < 0.05 was considered statistically significant.

DBP (mmHg)	Group A vs. Group B	
Time intervals	*r* mean	*P*-value
Baseline	0.533	0.847
2 minutes	9.66	0.009*
4 minutes	7.36	0.029*
6 minutes	6.96	0.036*
8 minutes	6.70	0.032*

## Discussion

In our study, thoracic segmental spinal anesthesia was given in the sitting position to both groups. We compared the impact of equal doses of hyperbaric and isobaric levobupivacaine on sensory block, motor block, hemodynamic changes, and adverse effects. The results indicated that hyperbaric levobupivacaine resulted in a faster onset and recovery of both sensory and motor blocks. In the hyperbaric group, peak sensory level (T4) reached faster, sufficient for the surgical procedure, and also sensory block was more reliable than the isobaric group. Also maximum Bromage scale was found in the group with hyperbaric levobupivacaine, leading to better surgeon and patient satisfaction scores. Due to the same reasons, surgical time was also shorter in the hyperbaric group.

For laparoscopic procedures, regional anesthesia has grown in popularity, especially for patients who are not candidates for general anesthesia. Imbelloni et al.'s recent research [6,7] has shown that TSA is a good substitute for both high-risk patients and healthy people. Regional anesthesia has several advantages over general anesthesia, including the preservation of airway reflexes, a lower dosage, an instant onset of action, an effective sensory and motor block, less blood loss, a lower risk of thrombosis, and the ability to identify problems early because the patient is still conscious during the procedure.

To lower the risk of nerve damage, the traditional method of spinal anesthesia, which Bier first proposed in 1898, usually entails puncturing the subarachnoid space (SAS), which is located well below the terminal of the spinal cord. In 2006, van Zundert started new research on segmental spinal anesthesia at the T10 level in a patient with severe lung disease, specifically for laparoscopic cholecystectomy [13].

Yousef et al. conducted a study comparing three anesthesia methods - segmental TSA (T10/T11), conventional lumbar spinal anesthesia (L2/L3), and general anesthesia in healthy patients scheduled for elective laparoscopic cholecystectomy. Their results showed that segmental TSA provides better hemodynamic stability, reduces the need for vasopressors, and allows for earlier walking and discharge, along with higher patient satisfaction. This makes TSA a great choice for daycare surgeries compared to conventional lumbar spinal anesthesia [14]. In a similar study, Bhattu et al. compared two groups of 50 patients each undergoing laparoscopic cholecystectomy. Group A received thoracic segmental spinal anesthesia at the T10 level with 1.75 mL (13.75 mg) of 0.5% isobaric levobupivacaine and 25 mcg of fentanyl, while Group B was given general anesthesia. The study concluded that thoracic segmental spinal anesthesia is a safe and effective alternative, providing excellent analgesia, and can be particularly beneficial for patients with comorbidities, allowing them to avoid general anesthesia [7].

The selection of local anesthetic agents for spinal anesthesia depends on various factors, including their efficacy in providing both intraoperative anesthesia and postoperative analgesia, while minimizing central and cardiovascular system side effects. Bupivacaine, a long-acting local anesthetic in the amino amide class, is commonly used in peripheral nerve blocks, and epidural, and spinal anesthesia. However, bupivacaine is associated with potential cardiovascular side effects due to its slow dissociation from sodium channels. This has led to the development of local anesthetics with similar properties to bupivacaine but with fewer cardiovascular and neurotoxic effects. Levobupivacaine, an enantiomer of bupivacaine, has been shown to have a comparable pharmacokinetic profile but with a lower risk of cardiotoxicity and neurotoxicity. Additionally, levobupivacaine demonstrates a faster protein binding rate, which contributes to a reduced degree of toxicity. Studies have compared hyperbaric levobupivacaine 0.5% with hyperbaric bupivacaine 0.5% for lower limb surgeries under spinal anesthesia and found both agents equally effective [15]. Furthermore, research by Kaur and Katoch comparing levobupivacaine and bupivacaine for TSA in laparoscopic cholecystectomy revealed that levobupivacaine had a slight advantage, providing a more prolonged sensory block. These findings suggest that levobupivacaine may offer a safer and more effective alternative to bupivacaine, particularly in TSA for laparoscopic procedures [16].

Baricity, which refers to the relative density of an intrathecal anesthetic solution compared to CSF, significantly impacts the distribution and extent of the subarachnoid block. Hyperbaric solutions generally provide a more predictable sensory block due to their tendency to settle in the dependent areas of the spinal column, thereby offering efficient analgesia during surgery. However, isobaric solutions, upon reaching the CSF, can act similarly to hypobaric solutions, which may lead to an upward migration of the block [17,18], potentially causing hemodynamic instability. As a result, hyperbaric solutions are often preferred in clinical practice because they provide a reliable block without the risk of excessive cephalad spread, thereby minimizing side effects and avoiding high block levels.

In line with this, a study by Sanansilp et al. compared the sensory effects of hyperbaric and isobaric levobupivacaine in 20 patients undergoing gynecological surgery. The results demonstrated that hyperbaric levobupivacaine produced a more predictable and consistent sensory block. In contrast, the isobaric form exhibited a broader range of peak sensory levels, extending from L1 to C8, while the hyperbaric solution's peak levels ranged from T7 to T2, indicating a more controlled block in the thoracic region [19]. Additionally, Şen et al. studied the effects of 13.5 mg of hyperbaric versus isobaric levobupivacaine in patients undergoing urological surgery. They found that the hyperbaric form resulted in a faster onset of both sensory and motor blocks, reached a higher maximum sensory block, and led to a quicker onset of Bromage score 3, indicating it was more effective in achieving motor blockade. However, both forms had similar times for the sensory block to wear off, with the hyperbaric group having a shorter duration for both sensory and motor blocks [11].

Further supporting the use of hyperbaric solutions, Kour et al. looked at how isobaric and hyperbaric bupivacaine affected hemodynamic parameters during thoracic combined spinal epidural anesthesia for laparoscopic cholecystectomy. Their study found very few neurological or postoperative complications, regardless of the type of bupivacaine used, showing that both forms are clinically safe [16,20].

While these findings suggest the advantages of hyperbaric solutions in providing a more predictable and controlled block, one limitation of our study is that we focused solely on levobupivacaine for laparoscopic cholecystectomy. Future research should consider evaluating the effects of levobupivacaine in longer, more painful surgeries, such as orthopedic procedures, to assess its efficacy and safety across a broader range of surgical settings. Additionally, studies could be designed to determine the minimum dose of levobupivacaine required to achieve effective sensory blockade while sparing motor function, enabling early postoperative ambulation and improving recovery times for patients. This could increase the advantages of segmental spinal anesthesia and offer a good alternative to general anesthesia for appropriate surgical cases. One limitation of our study is that we performed spinal anesthesia using levobupivacaine only for laparoscopic cholecystectomy. It is also necessary to perform similar studies for evaluation of the effects and duration of anesthesia provided by levobupivacaine in longer and more painful surgical procedures like orthopedic surgeries.

## Conclusions

The results revealed that in our study comparing isobaric levobupivacaine (0.5%) and hyperbaric levobupivacaine (0.5%) for thoracic segmental spinal anesthesia in laparoscopic cholecystectomy, the hyperbaric group demonstrated a faster onset and offset of both sensory and motor blocks. The sensory block reached T4 more quickly, and the maximum Bromage scale was achieved sooner in the hyperbaric group. Additionally, the hyperbaric group had better surgeon and patient satisfaction scores, along with a lower patient discomfort score. Although postoperative VAS scores were higher, the time to first rescue analgesia was shorter. Furthermore, hyperbaric levobupivacaine provided better hemodynamic stability, reduced hypotension, and led to a quicker recovery with fewer postoperative complications such as pneumonia and atelectasis. Future studies could further explore the long-term effects and recovery patterns of patients who undergo laparoscopic cholecystectomy with segmental thoracic spinal anesthesia using different formulations of local anesthetics. Research focusing on optimal dosing strategies, as well as potential combinations with adjunctive medications like opioids, would be valuable in refining pain management protocols.
